# Shoulder arthroplasty following a previous Latarjet procedure

**DOI:** 10.1016/j.jseint.2025.101609

**Published:** 2026-01-02

**Authors:** Matthias Biner, Bettina Hochreiter, Philipp Kriechling, Karl Wieser

**Affiliations:** Department of Orthopedics, Balgrist University Hospital, University of Zurich, Zurich, Switzerland

**Keywords:** Shoulder, Arthroplasty, Instability, Coracoid transfer, Bone block, Glenohumeral arthritis, Osteoarthritis

## Abstract

**Background:**

Shoulder arthroplasty in patients with prior Latarjet procedures is technically challenging due to altered anatomy and soft tissue changes. This study aimed to compare clinical outcome measures, complications, and implant survival in primary hemiarthroplasty (HA), anatomic total shoulder arthroplasty (aTSA), and reverse total shoulder arthroplasty (rTSA) in patients with previous Latarjet surgery vs. matched cohorts.

**Methods:**

A retrospective analysis was conducted including a total of 69 patients, comparing 23 post-Latarjet patients (4 HA, 6 aTSA, 13 rTSA) with a 2:1 matched control group. Outcomes included range of motion, functional scores (absolute and relative Constant-Murley Score [aCS and rCS], Subjective Shoulder Value), complications, revisions, and radiographic findings. Kaplan–Meier survival analysis evaluated reintervention-free survival.

**Results:**

Demographics were not significantly different between the post-Latarjet and matched cohorts. Patients who underwent HA following a Latarjet demonstrated significantly less improvement in flexion (Δ −10° vs. 50°), abduction (Δ 5° vs. 70°), and external rotation (Δ −15° vs. 30°) compared to their matched controls (all *P* < .05). Moreover, the improvement in aCS was significantly lower in the post-Latarjet HA group (13.5 vs. 36 points; *P* = .042). In the rTSA subgroup, flexion improvement was also significantly reduced in post-Latarjet patients compared to the matched cohort (Δ −10° vs. 37.5°; *P* = .018). Other functional outcomes (aCS, rCS, Subjective Shoulder Value) were comparable across groups. Complication rates were 75% (HA), 33.3% (aTSA), and 15.4% (rTSA) in the post-Latarjet group, compared to 25% (HA), 33.3% (aTSA), and 11.5% (rTSA) in the matched cohorts. Reintervention-free survival at 60 months was lowest for HA (25%), intermediate for aTSA (66.7%), and highest for rTSA (84.6%), with rTSA showing significantly better survival than HA (*P* = .009).

**Conclusion:**

While clinical outcomes were largely comparable between post-Latarjet patients and matched cohorts, rTSA may offer the most reliable results with lower complication and revision rates than aTSA and HA. It appears to be the preferred option in this complex setting, although post-Latarjet patients showed reduced range of motion.

Anterior shoulder dislocation represents the most common type of glenohumeral instability^12^ and frequently affects younger individuals.^22^ Recurrent instability progressively damages the glenohumeral joint, potentially leading to glenohumeral osteoarthritis (OA) if left untreated.^20^ Given the debilitating pain and functional limitations associated with OA,^26^ preventing joint deterioration remains a primary objective when treating anterior instability.^36^ Initial management typically involves nonoperative approaches;^10^ however, high recurrence rates^3^^,^^18^^,^^19^^,^^34^ often necessitate surgical intervention.^11^

Surgical management of anterior shoulder instability includes both arthroscopic and open procedures.^12^ In cases presenting significant glenoid bone loss, bone block procedures, including coracoid transfer procedures such as the Latarjet^24^^,^^38^ or Bristow^16^ procedures, are generally preferred.^28^

In the US, open Latarjet procedures increased by 250% from 2009 to 2018,^1^ or by 15% annually between 2007 and 2015 compared to an 8% annual increase in arthroscopic stabilization.^30^ Although effective for stability, coracoid transfers are associated with complications,^8^ including mid- to long-term OA.^27^ However, limited evidence regarding arthroplasty following osseous anterior stabilization procedures exists.^4^^,^^37^ The etiology of poststabilization OA remains incompletely understood, although glenohumeral general laxity, cartilage damage due to recurrent dislocations, or lateral overhang of a bone block have been proposed as potential risk factors.^21^ More clearly established is the correlation between OA and worse clinical outcome, particularly regarding pain and function,^2^ often necessitating surgical revision.^33^ The outcome of joint preserving revision procedures (ie, arthroscopic débridement, tenodesis of the long head of the biceps, and screw removal) is, however, not really predictable, and in case of severe joint degeneration prosthetic replacement might be considered or needed.

The purpose of this study was to evaluate shoulder arthroplasty after a previous Latarjet procedure by addressing two specific questions: (1) how different arthroplasty types (hemiarthroplasty (HA), anatomic total shoulder arthroplasty (aTSA), and reverse total shoulder arthroplasty (rTSA)) perform in post-Latarjet patients, and (2) how these outcomes compare to matched cohorts undergoing primary arthroplasty. We hypothesized that arthroplasty following a prior Latarjet procedure would be associated with higher complication and revision rates compared with matched primary arthroplasties, and that rTSA may provide more reliable results than aTSA or HA in this challenging setting.

## Material and methods

The study was approved by the Cantonal Ethics Committee Zurich (BASEC No. 2025-01414) and was conducted in accordance with the Declaration of Helsinki.^39^

### Study design

This retrospective matched cohort study was conducted at an academic tertiary referral center to investigate functional and radiographic outcomes across arthroplasty types (HA, aTSA, rTSA) in patients with prior Latarjet procedures and to compare these results with matched controls who underwent arthroplasty for primary glenohumeral OA or cuff tear arthropathy. For each post-Latarjet case, 2 controls were manually matched by age (±10 years), sex, body mass index (±5 points), prosthesis type, and available follow-up (±5 years). When several potential controls fulfilled these criteria, the case with the closest combination of age and follow-up duration was selected. Follow-up was comparable between groups, with small variations resulting from retrospective data availability. The study included 23 patients (4 HA, 6 aTSA, 13 rTSA) operated between May 2002 and January 2022 with a minimum follow-up of two years.

### Study cohort

Our study cohort comprised 23 patients (7 female, 30.4%) with a median age of 57 (interquartile range [IQR] 46.8, 62.4) at the time of surgery. Sixteen of the twenty-three patients (70%) had at least one previous shoulder operation before the Latarjet procedure, totaling 32 interventions (median 1 (IQR 0, 2); range 0-7). The most frequent surgeries were 13 Bankart repairs (10 open), followed by 5 derotational osteotomies, 3 rotator cuff reconstructions, 3 arthroscopies for infection, 2 bone block stabilizations, 2 rotator interval reconstructions, 2 capsular shifts, and 1 latissimus dorsi transfer. The median interval between the Latarjet and subsequent arthroplasty was 23.2 months (IQR 10.4, 55.4). Regarding surgical indications, 8 patients had glenohumeral OA, 5 had persistent instability, 7 had a combination of OA and instability, and 3 had cuff tear arthropathy. In the matched cohort, of the 8 HA patients, 2 had proximal humerus fractures, 2 had post-traumatic OA, 2 had irreparable rotator cuff lesions, and 2 had centered OA. Of the 12 aTSA patients, 7 had primary OA, and 5 had post-traumatic OA. Of the 26 rTSA patients, all had cuff tear arthropathy or an irreparable rotator cuff lesion.

### Surgical details

In 6 patients (26%), the Latarjet procedure had been performed at an external institution. For arthroplasty implantation, a deltopectoral approach was used in all patients. Intraoperatively, the quality of the subscapularis (SSC) was described as intact in 12 shoulders and scarred in 7 shoulders, while its condition was not explicitly noted in 4 cases. Joint exposure was achieved either via an SSC peel-off or, in 4 shoulders, through a lesser tuberosity osteotomy. Excessive intraoperative scarring was observed in 14 shoulders. Hardware removal was performed in all but one shoulder, with four shoulders having undergone hardware removal prior to the arthroplasty procedure. Intraoperative assessment revealed glenoid defects in 12 shoulders, with 6 receiving bony autograft augmentations for anterior glenoid bone loss—1 in HA, 1 in aTSA, and 4 in rTSA.

In the HA group, two shoulders received an anatomical stemmed prosthesis (Zimmer Biomet, Warsaw, IN, USA) and two EPOCA humeral cups (Synthes, West Chester, PA, USA). In the aTSA group, two SIDUS stemless (Zimmer Biomet, Warsaw, IN, USA), three anatomical (Zimmer Biomet, Warsaw, IN, USA), and one EPOCA (Synthes, West Chester, PA, USA) implants were used. The glenoid component was cemented in five of six aTSA cases. In the rTSA group, one Tornier long stem (Stryker, Kalamazoo, MI, USA), eleven anatomical reverse (Zimmer Biomet, Warsaw, IN, USA), and one Delta Xtend (Synthes, West Chester, PA, USA) were implanted. Postoperatively, all shoulders were immobilized in a sling for six weeks. Active-assisted mobilization was allowed on the first postoperative day, whereas active range of motion (ROM) as well as resistance exercises were implemented after 6 weeks.

### Clinical data

All patients at our institution who underwent arthroplasty are regularly invited for a clinical and radiographic follow-up six weeks postoperatively, 4.5 months, 2 years, 5 years, 10 years, and every five years thereafter. Pain assessment is conducted as part of the absolute Constant-Murley Score (aCS)^9^ using a 15 point Likert scale, where 15 points represents complete absence of pain. Global shoulder function, including activities of daily living, ROM, and abduction strength, is assessed with the aCS as well. All results are age-adjusted and presented as the relative CS (rCS).^9^ In addition, patient-reported outcomes are captured through the subjective shoulder value (SSV).^13^ These scores are assessed by the attending physicians during the clinical follow-up visits.

### Radiographic data

All radiographs were analyzed postoperatively and at regular follow-ups. Preoperative OA was graded according to Samilson and Prieto.^32^ Periprosthetic radiolucency was classified as none (grade 0), 1 mm and incomplete (grade 1), 1 mm and complete (grade 2), 1.5 mm and incomplete (grade 3), 1.5 mm and complete (grade 4), and 2 mm and complete (grade 5).^23^ For HA and aTSA, pre- and postoperative glenohumeral subluxation assessment involved measuring humeral head displacement relative to the center of the glenoid in the axial x-ray, with subluxation defined as displacement exceeding 25% translation. In addition, HA prostheses were assessed for glenoid erosion, classified as none, mild, moderate, or severe.^17^ Radiographic evaluation following rTSA included assessment of scapular notching using the Nerot–Sirveaux classification.^35^

### Statistical analysis

Summary statistics were presented as median and IQR for continuous data and as absolute and relative frequencies for categorical data. Functional outcome parameters were compared between groups using the Mann–Whitney *U* test, stratified by prosthesis type. Descriptive odds ratios for complication risk were calculated using Fisher exact test. Given the small sample size, all subgroup analyses are exploratory and no formal correction for multiple comparisons was applied. Statistical analysis was performed using MATLAB (2022b; MathWorks, Natick, MA, USA) and R/RStudio (version 4.4.2; Posit, Boston, MA, USA). *P* values < .05 were considered significant. Kaplan–Meier analysis was used to assess reintervention-free survival, and group differences were tested with the log-rank test.

## Results

No significant differences were observed in age, sex, body mass index, or time point of comparison between the post-Latarjet cohort and their matched groups (Table I). The post-Latarjet HA cohort demonstrated significantly poorer flexion, abduction, and external rotation compared to its matched group (all *P* < .05). In addition, the improvement in aCS was significantly lower in the post-Latarjet HA cohort (Δ 13.5 pts vs. Δ 36.0 pts; *P* = .042). The post-Latarjet aTSA cohort showed no statistically significant differences in ROM, aCS/rCS, or SSV when compared to its matched group. The post-Latarjet rTSA cohort demonstrated significantly poorer flexion compared to its matched group (median Δ −10.0° vs. Δ 37.5°; *P* = .018). No other significant differences in interindividual ROM, aCS/rCS, or SSV changes were observed (Tables II and III). Absolute pre- and postoperative values for ROM and functional outcomes are presented in Supplementary Tables SI and SII.

**Table I:** Demographic data.

	HA (Latarjet)	HA (matching)	aTSA (Latarjet)	aTSA (matching)	rTSA (Latarjet)	rTSA (matching)
Total (n)	4	8	6	12	13	26
Age at surgery (yr)	57.5 (53.5, 58.3)	57 (53.0, 59.5)	44.5 (39.8, 50.8)	44.5 (39.5, 50.3)	58 (48, 68)	60.5 (52.7, 65.9)
*P* value	n.s.		n.s.		n.s.	
Time point of comparison (mo)	57.9 (14.7, 103.7)	46.2 (25.6, 83.5)	42.6 (24, 111.7)	45.4 (25.1, 114.8)	24 (15.6, 60.0)	24.2 (23.9, 36.1)
*P* value	n.s.		n.s.		n.s.	
Sex (f/m)	1/3	2/6	1/5	2/10	5/8	10/16
*P* value	n.s.		n.s.		n.s.	
BMI	25.8 (23.8, 28)	25.6 (23.2, 33.1)	26.3 (24.3, 29.5)	26.4 (23.4, 29)	24.9 (20.2, 26.3)	24.5 (21.3, 26.1)
*P* value	n.s.		n.s.		n.s.	

*HA*, hemiarthroplasty; *aTSA*, anatomic total shoulder arthroplasty; *rTSA*, reverse total shoulder arthroplasty; *BMI*, body mass index; *IQR*, interquartile range; *n.s.,* not significant.

Values in median with IQR in parentheses.

**Table II:** ROM—absolute change from baseline (median, IQR).

	HA (Latarjet)	HA (matching)	aTSA (Latarjet)	aTSA (matching)	rTSA (Latarjet)	rTSA (matching)
Flexion (°)	−10 (−25, 7.5)	50 (32.5, 67.5)	0 (−20, 0)	40 (−10, 65)	−10 (−32.5, 35)	37.5 (−5, 60)
*P* value	**.006**		n.s.		.018	
Abduction (°)	5 (−7.5, 10)	70 (45, 91.3)	30 (0, 71.3)	15 (7.5, 87.5)	25 (−10, 60)	50 (30, 80)
*P* value	**.006**		n.s.		n.s.	
External rotation (°)	−15 (−47.5, −5)	30 (16.3, 40)	20 (10, 26.3)	12.5 (−10, 47.5)	−10 (−27.5, 2.5)	−10 (−15, 10)
*P* value	**.012**		n.s.		n.s.	
Internal rotation∗ (0-10)	1 (0, 3.0)	2 (−1.5, 4)	2 (−2, 2.5)	1 (−1, 5)	1 (−1, 2)	0 (−2, 5)
*P* value	n.s.		n.s.		n.s.	

*ROM*, range of motion; *HA*, hemiarthroplasty; *aTSA*, anatomic total shoulder arthroplasty; *rTSA*, reverse total shoulder arthroplasty; *IQR*, interquartile range; *n.s.,* not significant.

Absolute change from baseline (median, IQR); values in median with IQR in parentheses. Bold values indicate statistical significance (*P* < .05).

∗ Internal rotation rated on a 0-10 scale (10 = best outcome).

**Table III:** PROMs—absolute change (median, IQR).

	HA (Latarjet)	HA (matching)	aTSA (Latarjet)	aTSA (matching)	rTSA (Latarjet)	rTSA (matching)
aCS	13.5 (0.5, 24)	36 (24, 45)	20 (−3.5, 24.8)	18.5 (2, 49)	27 (6.5, 46.3)	27 (7, 41)
*P* value	**.042**		n.s.		n.s.	
rCS	17.3 (2.2, 25)	40 (25.4, 50.8)	20.4 (−1.9, 24.9)	22.1 (2.6, 57)	31 (6.1, 50.6)	28.9 (7.1, 45.3)
*P* value	n.s.		n.s.		n.s.	
SSV	55 (20, 87.5)	40 (26.3, 60.0)	35 (23.8, 50)	40 (−6.3, 53.8)	40 (16.3, 57.5)	35 (20, 50)
*P* value	n.s.		n.s.		n.s.	

*PROMs,* Patient-Reported Outcome Measures; *aCS*, absolute Constant-Murley Score; *rCS*, relative Constant-Murley Score; *SSV*, Subjective Shoulder Value; *HA*, hemiarthroplasty; *aTSA*, anatomic total shoulder arthroplasty; *rTSA*, reverse total shoulder arthroplasty; *IQR*, interquartile range; *n.s.,* not significant.

Absolute change (median, IQR); values in median with interquartile range in parentheses. Bold values indicate statistical significance (*P* < .05).

### Complications and revisions

**Figure 1:**
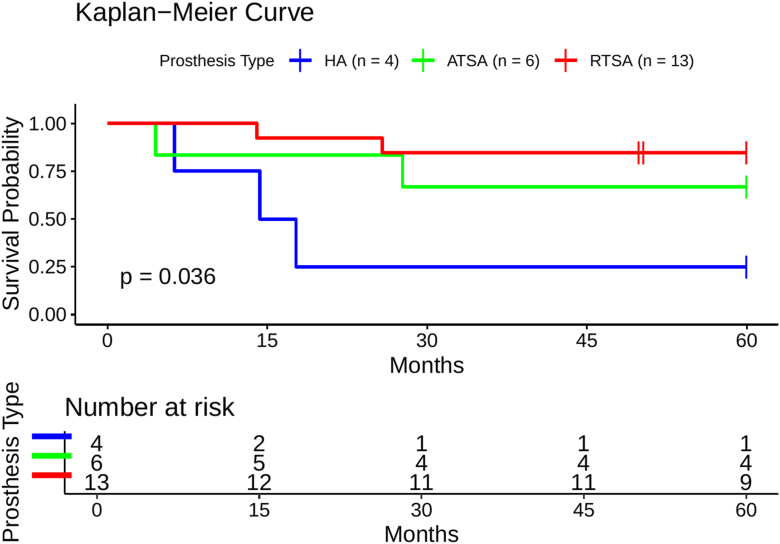
Kaplan–Meier survival curves showing reintervention-free survival by prosthesis type. Surgical reintervention was defined as the end point. The number at risk is displayed below the curves. HA = *blue*, aTSA = *green*, rTSA = *red*. Censored cases are indicated by tick marks. *HA*, hemiarthroplasty; *aTSA*, anatomic total shoulder arthroplasty; *rTSA*, reverse total shoulder arthroplasty.

Complications and revision rates at a maximum follow-up period of 10 years were analyzed for both post-Latarjet and matching groups, excluding trauma-related fracture cases. The complications were 75% vs. 25% for HA, 33.3% vs. 33.3% for aTSA, and 15.4% vs. 11.5% for rTSA in the post-Latarjet and matched groups, respectively (Table IV). Kaplan–Meier analysis showed a reintervention-free survival of 25.0%, 66.7%, and 84.6% for HA, aTSA, and rTSA, respectively (Fig. 1). Pairwise comparisons revealed significantly better survival for rTSA compared to HA (*P* = .009), while differences between rTSA and aTSA (*P* = .38) and between aTSA and HA (*P* = .20) were not significant.

**Table IV:** Complications and reintervention rates.

Prosthesis type	Group	Total complications	Complication rate (%)	Odds ratio	*P* value	Reintervention rate (%)
HA	Latarjet	3	75	7.22	n.s.	75
HA	Matching	2	25			25
aTSA	Latarjet	2	33.3	1	n.s.	33.3
aTSA	Matching	4	33.3			33.3
rTSA	Latarjet	2	15.4	1.38	n.s.	15.4
rTSA	Matching	3	11.5			0

*HA*, hemiarthroplasty; *aTSA*, anatomic total shoulder arthroplasty; *rTSA*, reverse total shoulder arthroplasty.

### Complications in the post-Latarjet groups

• HA: Three complications (75%) were observed among four patients, all of which required revision surgery. Two patients presenting with pain and functional impairment underwent conversion to aTSA, with intraoperative findings confirming advanced glenoid erosion. One patient with static anteroinferior subluxation was converted to rTSA.
• aTSA: Two complications (33.3%) among 6 patients, both requiring revision. One case underwent arthroscopic capsular plication for inferior subluxation, and another required conversion to rTSA for glenoid loosening and SSC insufficiency.
• rTSA: Two complications (15.4%) among 13 patients, both requiring revision. One case with persistent pain and functional impairment underwent arthroscopy and arthrolysis, while another case presenting with instability and external rotation deficit was managed with mobile component revision and muscle transfer (L'Episcopo).

In addition, two postoperative, trauma-related humeral shaft fractures were recorded in the post-Latarjet group undergoing rTSA (at 2 and 3 months postoperatively). One was treated with open reduction and internal fixation, and the other was managed conservatively. These cases were not counted toward the aforementioned prosthesis-related complications, as they were clearly trauma-related in origin.

### Radiographic analysis

**Figure 2:**
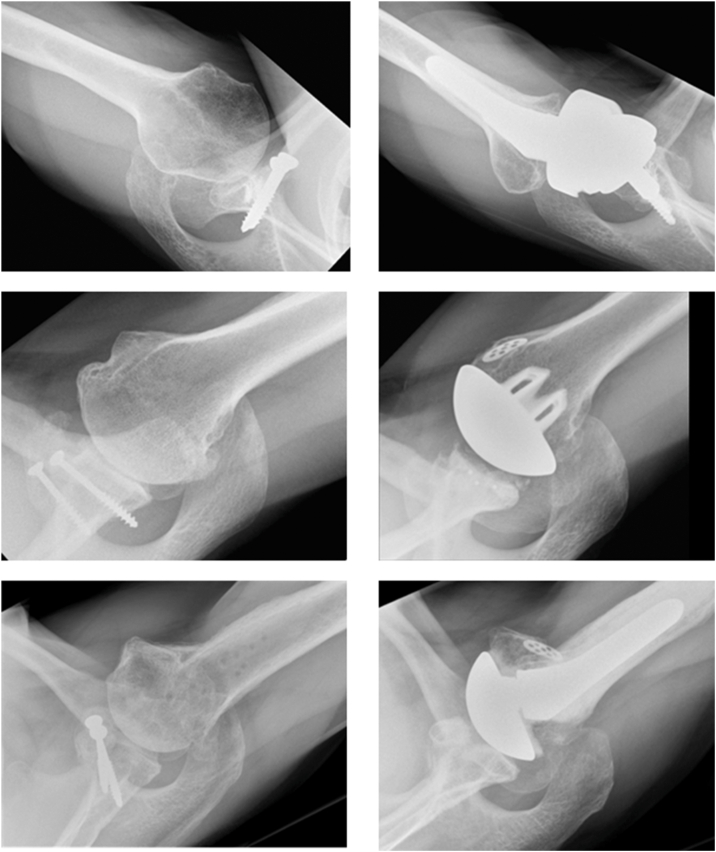
Representative axial X-rays obtained preoperatively (*left*) and postoperatively (*right*) after rTSA, aTSA, and HA procedures (*top to bottom*). The images illustrate the assessment of anterior subluxation and postoperative alignment used for radiographic analysis. *HA*, hemiarthroplasty; *aTSA*, anatomic total shoulder arthroplasty; *rTSA*, reverse total shoulder arthroplasty.

Among 4 HA cases, 3 showed preoperative anterior subluxation (75%), which persisted postoperatively in 2 (50%) (Fig. 2). Preoperative Samilson–Prieto grading showed grade 0 in 1 case (25%), grade I in 1 case (25%), and grade III in 2 cases (50%). No postoperative radiolucency was recorded.

Among the 6 aTSA cases, anterior subluxation was preoperatively observed in 5 cases (83.3%), which persisted in all of the patients postoperatively (Fig. 2). Preoperative Samilson–Prieto grading showed grade 0 in 1 case (16.7%), grade I in 2 (33.3%), grade II in 2 (33.3%), and grade III in 1 case (16.7%). Postoperative radiolucency was observed in three cases: one glenoidal (grade 1) and two humeral (one grade 1, one grade 2).

Among the 13 rTSA cases, preoperative anterior subluxation in axial X-rays was observed in 8 patients (61.5%) (Fig. 2). Preoperative Samilson–Prieto grading showed grade 0 in 2 (15.4%), grade I in 3 (23.1%), grade II in 6 (46.2%), and grade III in 1 (7.7%), with 1 case not applicable (7.7%). Postoperative periprosthetic humeral radiolucency grade 1 occurred in 3 (23.1%). Notching was present in 61.5% of all cases: grade 0 in 5 (38.5%), grade 1 in 3 (23.1%), grade 2 in 4 (30.8%), and grade 3 in 1 (7.7%).

Preoperative anterior subluxation was present in 16 of 23 patients (69.6%), with complications occurring in 5 of these 16 cases (31.3%), compared to 2 of 7 (28.6%) without subluxation. In aTSA, patients with subluxation tended to experience both more complications and greater functional improvement (mean ΔaCS +26.5 vs. 0). Given the small group sizes, these findings should be regarded as exploratory. No consistent association was observed between Samilson–Prieto grade and functional outcomes or complication rates within the post-Latarjet group.

## Discussion

The most important finding of this study was that, while objective and subjective outcomes were comparable between the post-Latarjet and matched cohorts across most comparisons, rTSA may offer the most predictable outcomes with lower complication and revision rates compared to TSA and HA in this population. Notably, in the HA subgroup, several objective measures such as ROM and CS were lower in post-Latarjet patients compared with matched controls.

While only two studies^4^^,^^37^ have specifically examined arthroplasty following isolated coracoid transfer, a limited number of studies have analyzed mixed stabilization procedures.^5^^,^^15^^,^^25^^,^^29^ Our findings align with those of previous studies. Willemot et al^37^ reported a complication rate of 42.1% across all prosthesis types, with HA showing the highest complication rate (66.6%) among 3 patients, aTSA showing 44.4% among 9 patients, and rTSA demonstrating the lowest (28.6%) among 7 patients. Revisions in their HA and aTSA cohorts were primarily due to instability and glenoid loosening, while their rTSA had no recorded revisions. Similarly, Bender et al^4^ found that while aTSA achieved satisfactory functional outcomes, it demonstrated a complication rate of 27.2% among 11 patients and a revision rate of 18.2%, attributable to glenoid loosening and instability. These studies, alongside our findings, emphasize the technical challenges and variability in outcomes associated with different arthroplasty options in the post-Latarjet population. In our cohort, rTSA was associated with the lowest complication and revision rates, whereas HA showed the highest. While the difference between rTSA and aTSA was not statistically significant, the consistency of better outcomes with rTSA across multiple studies suggests that prosthesis selection may play an important role in this specific patient group.

In the aTSA group, preoperative subluxation appeared to be associated with both higher complication rates and greater functional improvement, whereas this trend was not observed in rTSA or HA. Although these findings suggest a possible link between radiographic alterations and clinical outcomes, they should be interpreted with caution given the small subgroup sizes.

In this study, patients undergoing HA and rTSA post-Latarjet demonstrated significantly poorer postoperative ROM compared to the matched group. Interestingly, these patients lost approximately 10° of flexion and external rotation compared to preoperatively, while their matched groups gained flexion and external rotation (only HA). This may be attributed to the fact that patients post-Latarjet had better ROM to begin with and less potential for improvement. Furthermore, the matched group of rTSA had more potential for improvement in flexion, as these patients typically presented with a nonfunctioning rotator cuff. However, overall functional outcomes, including CS and SSV, were comparable across most groups, with the exception of HA, where the post-Latarjet group exhibited significantly worse improvement in aCS (but not rCS).

In our post-Latarjet cohort, complication and revision rates were highest for HA (75%), intermediate for aTSA (33.3%), and lowest for rTSA (15.4%). This pattern was consistent in the matched cohorts (25%, 33.3%, and 11.5%, respectively), supporting the greater reliability of rTSA in this challenging setting. The more consistent results after rTSA may be related to its higher intrinsic constraint and deltoid-dependent stability compared with anatomic designs. Its semiconstrained design increases compressive joint forces and soft-tissue tension, providing stability without the need for extensive soft-tissue balancing and compensating for instability,^6^^,^^7^ SSC deficiency, and capsular scarring commonly seen after coracoid transfer. These biomechanical mechanisms have been shown to improve joint stability and load distribution in reverse constructs.^14^^,^^31^ The disparity in follow-up periods reflects the evolution of our institutional approach to post-Latarjet arthroplasty. Initially, hemiarthroplasty or aTSA was the preferred option, but our practice has shifted predominantly toward rTSA in recent years due to the high complication rates observed with anatomic implants. While our data suggests comparable revision-free survival times across implant types, comprehensive long-term follow-up data for rTSA is necessary before drawing definitive conclusions. In our experience, aTSA remains a valuable alternative only for carefully selected cases meeting specific criteria: preserved bone stock, functional rotator cuff (especially SSC), and a centered glenohumeral joint without evidence of persistent instability.

### Limitations

This study has several limitations. As a tertiary referral center, our patient population may represent more complex cases, introducing potential selection bias. The retrospective design introduces potential selection and information bias. The small sample size, particularly in the HA and aTSA subgroups, limits statistical power and precision of the estimates. Consequently, odds ratios and *P* values should be interpreted as descriptive and exploratory. No formal adjustment for multiple testing was performed. Heterogeneity in surgical indications and prior procedures between post-Latarjet and matched cohorts may have introduced confounding. In addition, the extensive surgical history of many post-Latarjet patients may have contributed to soft-tissue scarring, SSC insufficiency, and glenoid bone loss, potentially influencing both implant selection and postoperative outcomes. Multiple implant systems were used across arthroplasty types, which may limit generalizability. Nevertheless, the presented data provide meaningful mid-term insights, and larger cohorts with long-term follow-up are needed to confirm these findings.

## Conclusion

While overall outcomes were comparable, the post-Latarjet group showed reduced ROM in rTSA and both lower ROM and CS gains in HA compared with matched controls. rTSA remained the most reliable option, with lower complication and revision rates than aTSA and HA.

## Disclaimers:

Funding: No funding or grants were obtained for this study.

Conflicts of interest: The authors, their immediate families, and any research foundations with which they are affiliated have not received any financial payments or other benefits from any commercial entity related to the subject of this article.
