# Supplementary material for: Shoulder arthroplasty following a previous Latarjet procedure
Source: JSES Int. 2026 Jan 2;10(2):101609. doi: 10.1016/j.jseint.2025.101609 (PMC12925336; doi:10.1016/j.jseint.2025.101609)
Supplement: Supplementary Table S1 [file mmc1.docx]

**Supplementary Table I: Range of Motion (ROM) – pre- and postoperative values**

|  | **HA preop (Latarjet)** | **HA preop (Matching)** | **HA postop (Latarjet)** | **HA postop (Matching)** |
| --- | --- | --- | --- | --- |
| **Flexion (°)** | 125  (72.5, 155) | 90  (60, 105) | 100  (65, 145) | 145  (90, 160) |
| **p-value** | n.s. | | n.s. | |
| **Abduction (°)** | 115  (70, 155) | 80  (48.8, 87.5) | 115  (67.5, 160) | 145  (90, 160) |
| **p-value** | n.s. | | n.s. | |
| **External Rotation (°)** | 40  (35, 50) | 10  (-7.5, 27.5) | 25  (-12.5, 45) | 35  (30, 50) |
| **p-value** | **0.018** | | n.s. | |
| **Internal Rotation* (0–10)** | 4  (0, 9) | 6  (2, 6) | 6  (3, 9) | 6  (5, 6) |
| **p-value** | n.s. | | n.s. | |

|  | **aTSA preop (Latarjet)** | **aTSA preop (Matching)** | **aTSA postop (Latarjet)** | **aTSA postop (Matching)** |
| --- | --- | --- | --- | --- |
| **Flexion (°)** | 135  (120, 140) | 97.5  (75, 130) | 130  (112.5, 135) | 140  (115, 160) |
| **p-value** | n.s. | | n.s. | |
| **Abduction (°)** | 105  (80, 120) | 75  (55, 145) | 150  (123.8, 155) | 152.5  (100, 160) |
| **p-value** | n.s. | | n.s. | |
| **External Rotation (°)** | 35  (20, 50) | 10  (0, 30) | 60  (47.5, 65) | 25  (10, 60) |
| **p-value** | **0.042** | | n.s. | |
| **Internal Rotation* (0–10)** | 4  (2, 6) | 5  (2, 7) | 8  (4, 8.5) | 6  (6, 7) |
| **p-value** | n.s. | | n.s. | |

|  | **rTSA preop (Latarjet)** | **rTSA preop (Matching)** | **rTSA postop (Latarjet)** | **rTSA postop (Matching)** |
| --- | --- | --- | --- | --- |
| **Flexion (°)** | 95  (82.5, 130) | 90  (60, 150) | 105  (90, 120) | 132.5  (110, 145) |
| **p-value** | n.s. | | **0.027** | |
| **Abduction (°)** | 80  (60, 90) | 60  (50, 90) | 110  (80, 132.5) | 142.5  (100, 155) |
| **p-value** | n.s. | | n.s. | |
| **External Rotation (°)** | 40  (17.5, 50) | 40  (15, 50) | 20  (10, 37.5) | 30  (20, 40) |
| **p-value** | n.s. | | n.s. | |
| **Internal Rotation* (0–10)** | 4  (2, 7) | 6  (2, 6) | 6  (2, 8) | 4  (2, 8) |
| **p-value** | n.s. | | n.s. | |

Range of Motion (ROM) – pre- and postoperative values; Values in median with interquartile range (IQR) in parentheses. *Internal rotation rated on a 0–10 scale (10 = best outcome). Preop: preoperative; postop: postoperative; HA: Hemiarthroplasty; aTSA: Anatomic Total Shoulder Arthroplasty; rTSA: Reverse Total Shoulder Arthroplasty
