# Supplementary material for: Shoulder arthroplasty following a previous Latarjet procedure
Source: JSES Int. 2026 Jan 2;10(2):101609. doi: 10.1016/j.jseint.2025.101609 (PMC12925336; doi:10.1016/j.jseint.2025.101609)
Supplement: Supplementary Table S2 [file mmc2.docx]

**Supplementary table II: Patient-Reported Outcome Measures (PROMs) – pre- and postoperative values**

|  | **HA preop (Latarjet)** | **HA preop (Matching)** | **HA postop (Latarjet)** | **HA postop (Matching)** |
| --- | --- | --- | --- | --- |
| **aCS** | 42.5  (17, 69) | 34  (21.5, 36) | 53.5  (39.5, 71) | 67  (55, 77) |
| **p-value** | n.s. | | n.s. | |
| **rCS** | 47.9  (21, 76) | 40  (28, 67.6) | 57.8  (44.8, 79.4) | 79.7  (66.2, 89.3) |
| **p-value** | n.s. | | n.s. | |
| **SSV** | 15  (5, 20) | 30  (22.5, 50) | 67.5  (35, 97.5) | 80  (70, 90) |
| **p-value** | n.s. | | n.s. | |

|  | **aTSA preop (Latarjet)** | **aTSA preop (Matching)** | **aTSA postop (Latarjet)** | **aTSA postop (Matching)** |
| --- | --- | --- | --- | --- |
| **aCS** | 40.5  (32, 64) | 41.5  (23, 62.5) | 66  (49, 72) | 69.5  (56, 78.5) |
| **p-value** | n.s. | | n.s. | |
| **rCS** | 44.1  (34, 68.8) | 45.1  (31, 66.6) | 69.6  (60.6, 76.8) | 76.1  (64.8, 89.9) |
| **p-value** | n.s. | | n.s. | |
| **SSV** | 45  (10, 50) | 40  (15, 50) | 70  (63.8, 88.8) | 60  (50, 87.5) |
| **p-value** | n.s. | | n.s. | |

|  | **rTSA preop (Latarjet)** | **rTSA preop (Matching)** | **rTSA postop (Latarjet)** | **rTSA postop (Matching)** |
| --- | --- | --- | --- | --- |
| **aCS** | 33  (21.5, 37) | 31  (23, 45) | 59  (44.3, 66.8) | 68.5  (40, 77) |
| **p-value** | n.s. | | n.s. | |
| **rCS** | 37.4  (26.5, 42.2) | 41.9  (29, 56) | 68  (48, 79.1) | 79.3  (42.5, 91.9) |
| **p-value** | n.s. | | n.s. | |
| **SSV** | 30  (20, 30) | 20  (10, 50) | 70  (45, 87.5) | 70  (40, 95) |
| **p-value** | n**.s.** | | n.s. | |

Functional outcomes – pre- and postoperative values; Values in median with interquartile range (IQR) in parentheses. Comparison of functional scores between patients after a prior Latarjet procedure and matched controls. P-values represent group comparisons (Latarjet vs Matching) for pre- and postoperative timepoints. aCS: absolute Constant Score; rCS: relative Constant Score; SSV: Subjective Shoulder Value; HA: Hemiarthroplasty; aTSA: Anatomic Total Shoulder Arthroplasty; rTSA: Reverse Total Shoulder Arthroplasty.
